# Genetic Characterization and Population Connectedness of North American and European Dairy Goats

**DOI:** 10.3389/fgene.2022.862838

**Published:** 2022-06-17

**Authors:** Marc Teissier, Luiz F. Brito, Flavio S. Schenkel, Guido Bruni, Pancrazio Fresi, Beat Bapst, Christèle Robert-Granie, Hélène Larroque

**Affiliations:** ^1^ GenPhySE, Université de Toulouse, Toulouse, France; ^2^ Department of Animal Sciences, Purdue University, West Lafayette, IN, United States; ^3^ Department of Animal Biosciences, Centre for Genetic Improvement of Livestock, University of Guelph, Guelph, ON, Canada; ^4^ ARAL, Crema, Italy; ^5^ AssoNaPa, Roma, Italy; ^6^ Qualitas AG, Zug, Switzerland

**Keywords:** dairy goats, genetic diversity, population structure, small ruminants, Alpine goats, Saanen goats

## Abstract

Genomic prediction of breeding values is routinely performed in several livestock breeding programs around the world, but the size of the training populations and the genetic structure of populations evaluated have, in many instances, limited the increase in the accuracy of genomic estimated breeding values. Combining phenotypic, pedigree, and genomic data from genetically related populations can be a feasible strategy to overcome this limitation. However, the success of across-population genetic evaluations depends on the pedigree connectedness and genetic relationship among individuals from different populations. In this context, this study aimed to evaluate the genetic connectedness and population structure of Alpine and Saanen dairy goats from four countries involved in the European project SMARTER (SMAll RuminanTs Breeding for Efficiency and Resilience), including Canada, France, Italy, and Switzerland. These analyses are paramount for assessing the potential feasibility of an across-country genomic evaluation in dairy goats. Approximately, 9,855 genotyped individuals (with 51% French genotyped animals) and 6,435,189 animals included in the pedigree files were available across all four populations. The pedigree analyses indicated that the exchange of breeding animals was mainly unilateral with flows from France to the other three countries. Italy has also imported breeding animals from Switzerland. Principal component analyses (PCAs), genetic admixture analysis, and consistency of the gametic phase revealed that French and Italian populations are more genetically related than the other dairy goat population pairs. Canadian dairy goats showed the largest within-breed heterogeneity and genetic differences with the European populations. The genetic diversity and population connectedness between the studied populations indicated that an international genomic evaluation may be more feasible, especially for French and Italian goats. Further studies will investigate the accuracy of genomic breeding values when combining the datasets from these four populations.

## 1 Introduction

Genomic prediction of breeding values is routinely performed in several livestock species, including dairy and beef cattle, dairy sheep, and dairy goats (Boichard et al., 2012; Carillier et al., 2013; Baloche et al., 2014; Ibanez-Escriche and Simianer, 2016; Rupp et al., 2016). Genomic selection has become possible due to the availability of a large enough training population (individuals with both genotypes and phenotypes for the traits of interest) genotyped for thousands of genomic markers. However, the success of these genomic predictions depends on population-specific parameters, including the effective population size, level of linkage disequilibrium (LD), genetic relationship between the training and target populations, pedigree connectedness, and trait heritability (Misztal et al., 2020; van den Berg et al., 2020; VanRaden, 2020). For instance, a single-nucleotide polymorphism (SNP) chip panel of enough SNP density is required to capture the LD between quantitative trait loci (QTL) and surrounding markers and thus accurately estimates the SNP effects (de Roos et al., 2008; Lund et al., 2011). The size of the training populations and the pedigree connectedness also play a major role in the accuracy of genomic predictions (Lund et al., 2011; VanRaden, 2020), and lower-heritability traits require an even larger training population (Pszczola et al., 2012).

Combining data from genetically related populations can be an efficient strategy for enlarging training populations for genomic predictions (Berry et al., 2014; Cardoso et al., 2021). For instance, this has been performed in European dairy cattle populations through the Eurogenomics Consortium (www.eurogenomics.com/), which maintains a training population of ∼40,000 genotyped bulls and provides genomic estimated breeding values (GEBVs) for 11 countries. More recently, international genomic evaluations have also been implemented in beef cattle populations (Bonifazi et al., 2020). In general, the method chosen to conduct these analyses is the multi-trait single-step genomic best linear unbiased prediction [ssGBLUP (Bonifazi et al., 2020)], in which the same trait measured across countries is considered different, but genetically correlated, traits. International genomic evaluations have been successfully implemented in international beef and dairy cattle populations. However, the success of across-population genomic evaluations requires a close collaboration between the partners and close population structure and genetic connectedness among the involved populations. For instance, the level of genetic connectedness (as a consequence of the exchange of genetic material) between the different populations needs to be sufficient to obtain accurate genomic prediction (Weigel et al., 2000; Fouilloux et al., 2006). Furthermore, combining data from several populations is only feasible if they are genetically related (Lund et al., 2014; Rezende et al., 2020). However, recent studies in Norwegian and New Zealand sheep with similar development history, but reduced recent exchange of genetic material, have reported that collaborative genomic analyses could still be feasible (Oliveira et al., 2020; Oliveira et al., 2022).

Currently, genomic evaluations have been implemented in dairy goats in France (Carillier et al., 2013) and tested in Canada (Massender et al., 2022) for both Alpine and Saanen breeds. GEBVs are more accurate than pedigree-based EBVs (Carillier et al., 2013; Carillier et al., 2014; Massender et al., 2022), but the observed gains in accuracy are still lower than dairy cattle. This is likely due to specific population characteristics such as the smaller size of the training populations and higher genetic diversity in dairy goats (Carillier et al., 2013; Brito et al., 2015). Combining data from different countries could contribute to improving the accuracy of genomic predictions by increasing the size of the training populations for economically important traits. Furthermore, across-country genomic predictions could be even more beneficial to countries that do not currently carry out genomic evaluations, such as Italy and Switzerland. Therefore, there is a need to assess the genetic connectedness and population structure of dairy goats from France, Italy, Canada, and Switzerland to evaluate the feasibility of an across-country genomic evaluation. In this context, the main objectives of this study were 1) to investigate the historical exchanges of genetic material between these four countries based on pedigree recording (genetic connectedness) and 2) to evaluate the genomic relatedness of these four populations based on genome-wide levels of LD, consistency of the gametic phase across population pairs, principal component analysis (PCA), and population admixture analyses. These analyses are paramount for assessing the potential feasibility of an across-country genomic evaluation in dairy goats.

## 2 Materials and Methods

### 2.1 Pedigree and Genomic Datasets

This study was carried out in the framework of the “practical selection tools to benefit from international harmonisation and cooperation” work package of the SMARTER project (www.smarterproject.eu/). Four countries (Canada, France, Italy, and Switzerland) have shared 9,941 raw genotypes and pedigree information from Alpine and Saanen dairy goat populations. The animal identification (ID) was standardized in each country partner and was formed based on four components: three letters indicating the breed of the animal (ALP for Alpine and SAA for Saanen) + three letters indicating the country of origin (CAN, FRA, ITA, and CHE representing Canada, France, Italy, and Switzerland, respectively) + one letter indicating the sex of the animal (F for female and M for male) + 16 characters with the animal identifier (including the animal birth country code in two letters and the remaining characters after adding the animal ID completed on the left side by as many 0 as needed). For instance, the final identification of an Alpine female with local ID 5248383, born in France, and raised in Switzerland would be ALPCHEF0000000FR5248383. Imported animals may have multiple identifiers (one from the country of origin and another one in the importing country). Therefore, up to three aliases could be provided by the partners in addition to the ID of the animal. This identification is important to enable tracing the origin of the curated data but also useful for finding the connections between the different pedigrees.

Various quality control filters were implemented in these datasets. First, the format of each animal’s identification included in the pedigree files was verified for consistency, including checking that all the animals present as sires or dams were also registered as individuals in the pedigree. After removing or correcting these inconsistencies, 6,435,189 animals remained in the pedigree files (Table 1). The pedigree file had 86%, 89%, 91%, and 94% females in Canada, Switzerland, France, and Italy, respectively, which were born between 1944 and 2020. The males were born from 1936 to 2020.

**TABLE 1:** Number of animals after the quality control, per breed (Alpine and Saanen), included in the pedigree and genotype files shared by each country (Canada, France, Italy, and Switzerland).

Country	Alpine		Saanen			
	Pedigree	Genotypes	Pedigree	Genotypes	Total pedigree	Total genotype
Canada	56,601	793	36,741	903	93,342	1,696
France	3,518,473	2,968	2,527,443	2,009	6,045,916	4,977
Italy	107,566	1,061	131,376	338	238,942	1,399
Switzerland	28,083	1,280	28,906	503	56,989	1,783
Total	3,710,723	6,102	2,724,466	3,753	6,435,189	9,855

All the individuals were genotyped using the same SNP chip panel, i.e., Goat SNP50 BeadChip (Illumina Inc., San Diego, CA, United States). There are currently two versions of this SNP chip panel, but 90% of the genotyping was performed based on the first version that contains 53,347 SNPs. Genotypes were exchanged in the TOP/BOT format and based on the ARS1 reference genome. As more than 90% of the genotyping was carried out based on the first version of the SNP chip panel and all SNPs included in the version 1 (*n* = 53,347) were also present in version 2, only the SNPs from version 1 were considered for further analyses. Duplicated genotypes were filtered out based on the animal call rate, in which the genotype sample with a higher call rate was kept in the dataset. SNPs with a minor allele frequency (MAF) lower than 0.01 and a call rate lower than 0.90 were filtered out. Furthermore, animals with a sample call rate lower than 0.90 were also removed from the analyses (*n* = 86). Quality control was performed within the breed and country but also after merging the four datasets. The quality control analyses were performed by PLINK 1.9 software (Purcell et al., 2007). After quality control, 9,855 animals and 50,578 SNPs remained for further analyses (Table 1).

**FIGURE 1:**
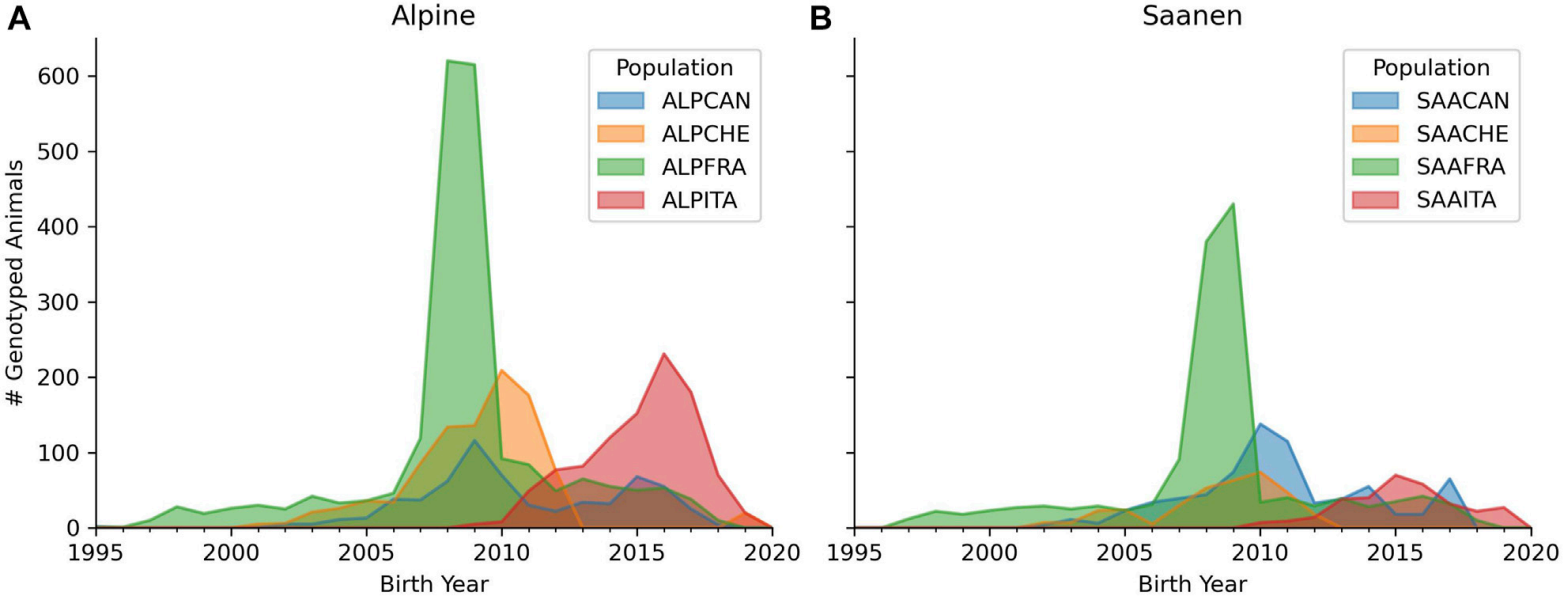
Number of genotyped animals according to the birth year for Alpine **(A)** and Saanen **(B)** breeds in each country (France, Canada, Italy, and Switzerland). The legend represents the breed (ALP for Alpine and SAA for Saanen) and country (CAN for Canada, CHE for Switzerland, FRA for France, and ITA for Italy).

Figure 1 shows a density plot of the birth years of Alpine- and Saanen-genotyped animals in each country. An important point to highlight is that the genotyping activities did not start at the same time across partners. The oldest genotyped animals were born in 1997, 2000, 2001, and 2009 for French, Swiss, Canadian, and Italian goats, respectively.

### 2.2 Pedigree Connectedness

The pedigree connection evaluations were conducted by pairs of countries, comparing a source pedigree and a target pedigree. The goal was to extract animals from the source country in the target pedigree and seek to find them in the source pedigree (for example, French animals from the Swiss pedigree were found in the French pedigree). In total, 12 comparisons were made to find all the connections. These analyses were performed using Python scripts prepared by the authors.

The standardization of animal identification facilitated the extraction of foreign animals present in the other pedigree files. Several strategies were developed to retrieve the pedigrees of these animals. The simplest approach was to compare the identifiers (and aliases) of these animals with the source pedigree (e.g., France). This step was easily automated but not sufficient to find all the pedigree connections. For instance, considering the Swiss dairy goat pedigree, some French animals were registered in Switzerland with only the last digits of the French identifiers. For these animals, we used the fuzzy string-matching approaches (with the fuzzywuzzy library in Python; https://pypi.org/project/fuzzywuzzy/) to find the matches between the two pedigrees. The verification of the proposed animal matches based on this approach was carried out manually. This approach enabled the identification of animals with typos at the time of registration.

### 2.3 Characterization of Genetic Diversity

#### 2.3.1 Linkage Disequilibrium

The extent of LD was calculated for each breed both within each country and also with merged datasets. This was determined based on the −r^2^ option implemented in the PLINK 1.9 software (Purcell et al., 2007). The r^2^ statistic was calculated as $\frac{{\left(p_{AB}-p_{A}p_{B}\right)}^{2}}{pA\left(1-p_{1}\right)p_{b}\left(1\;-\;p_{B}\right)}$ , where p_A_ and p_B_ are the respective frequencies of alleles A and B (two different loci), respectively, and p_AB_ is the frequency of the haplotype AB, as proposed by Hill and Robertson (1968). The LD between markers was measured for each pair of SNPs within a chromosome. The distance between two SNPs ranging from 0 to 1 Mb was categorized into 50 classes of 20 kb. The average LD was obtained by calculating the average r^2^ for each class. In the Sections 3, 4, each class was named based on the median distance in each interval. The LD decay plots were also created for each breed within the country.

#### 2.3.2 Consistency of the Gametic Phase

The calculation of consistency of the gametic phase was determined following Oliveira et al. (2020) by first calculating the square roots of the r^2^ statistic and then adding the sign of the D-value obtained with the dprime-signed option of the PLINK 1.9 software (Purcell et al., 2007). The consistency of the gametic phase was obtained as the Pearson correlation coefficient calculated between the signed-squared-root values of each country pair within the breed and between the signed-squared-root values across the two breeds within the country when grouping the two breeds together. The consistency of the gametic phase was also calculated for nine categories of SNPs according to their distance: (0 kb, 1 kb], (1 kb, 10 kb], (10 kb, 20 kb], (20 kb, 40 kb], (40 kb, 60 kb], (60 kb, 100 kb], (100 kb, 200 kb], (200 kb, 500 kb], and (500 kb, 1,000 kb]. We used the same interval classes as those presented by Mdladla et al. (2016).

#### 2.3.3 Inbreeding Estimation

In addition to linkage disequilibrium and consistency of the gametic phase, we investigated inbreeding of genotyped animals. Pedigree-based inbreeding was estimated by inbupgf90 software (Misztal et al., 2002). Genomic inbreeding was estimated in two steps by PLINK 1.9 software (Purcell et al., 2007). The first step was to prune SNPs with the options –indep; then, inbreeding was estimated on the prune dataset with the options –het and –ibc.

#### 2.3.4 Genetic Relatedness and Population Structure Analyses

The study of the genetic similarity and structure of the eight populations (two breeds x four countries) was performed based on two methods: principal component analysis (PCA) and genetic admixture analysis. To comply with the data independence assumption for performing PCA, the genotypes were pruned using the default parameter of the option -indep implemented in the PLINK 1.9 software (Purcell et al., 2007). A total of 31,951 SNPs were retained for the PCA analyses. PCA was performed using the -pca option of the PLINK 1.9 software (Purcell et al., 2007). The PCA was applied to the matrix of genomic relationships calculated as in Yang et al. (2011). The same pruned dataset was used to perform the admixture analysis using the Admixture software (Alexander et al., 2009). This software clusters individuals into k predefined groups according to allele frequencies (Oliveira et al., 2020). We tested k values ranging from 2 to 8 as it would be a more representative value of the expected number of subpopulations in our dataset. Only results with a k value equal to 4 will be presented because it yielded the lowest cross-validation error.

## 3 Results

### 3.1 Pedigree Connectedness

Table 2 describes the animals registered in several pedigrees for pairwise pedigree comparisons based on the animals’ country of origin. Some animals could be identified as belonging to a country, but their pedigrees were not found in the country of origin. This scenario corresponds to the row “missing in local pedigree” in Table 2. We observed that only French and Swiss animals were found in several pedigree files. French animals were found in all pedigrees (Canada, France, Italy, and Switzerland), indicating that France exported animals to all country partners of the project. However, France did not import any animals from these countries. In contrast, Italian and Canadian animals were not exported to any other country based on the available recording. The pedigree comparison of these two countries shows that they have only French animals in common, which are all found in the French pedigree: 94 Alpine and 41 Saanen (Table 2). Italy was the only country that imported animals from both France (9,037 animals) and Switzerland (1,095 animals). In Italy, 1,863 French animals were not found in the French pedigree (859 Alpine and 1,004 Saanen). This number corresponds to 309 for French animals in Switzerland and 495 for Swiss animals in Italy.

**TABLE 2:** Pedigree connectedness for Alpine and Saanen populations between pairs of four countries. Country abbreviations are CAN for Canada, CHE for Switzerland, FRA for France, and ITA for Italy. The native country is provided in each animal’s name; it is possible to check if a foreign animal in a pedigree is found in its native pedigree (found in local pedigree) or not (missing in local pedigree).

Pairwise pedigree comparisons									
Status	Local origin of animal	Breed	CAN-FRA	CAN-ITA	CAN-CHE	FRA-ITA	CHE-FRA	CHE-ITA	All
Found in local pedigree	CHE	ALP	0	0	0	0	0	798	798
	CHE	SAA	0	0	0	0	0	297	297
	FRA	ALP	119	94	25	4,580	187	138	5,143
	FRA	SAA	61	41	9	4,457	135	85	4,788
Missing in local pedigree	CHE	ALP	0	0	0	0	0	305	305
	CHE	SAA	0	0	0	0	0	190	190
	FRA	ALP	0	0	0	859	215	0	1,074
	FRA	SAA	0	0	0	1,004	94	0	1,098
		All	180	135	34	10,900	631	1,813	13,693

Since the majority of animal exchanges occurred between France and the other three countries, we have identified Canadian, Italian, and Swiss animals with French parents to estimate the importance of their descendants in the host country. Table 3 presents the number of Canadian, Italian, and Swiss animals with a French sire. In total, 17,137 Italian animals had a French sire, which represented 7.2% of the Italian pedigree. This proportion was lower for the Swiss (1.54%) and Canadian (0.58%) populations. For animals with a French dam, we observed lower numbers: 3,932 (1.6%) Italian animals, 101 (0.1%) Swiss animals, and 1 (0.0%) Canadian animals.

**TABLE 3:** Number of Canadian (CAN), Italian (ITA), and Swiss (CHE) animals with a French (FRA) sire for the Alpine (ALP) and Saanen (SAA) breeds according to the sex of the animals (M for male and F for female). The proportion of animals relative to the native pedigree is given in parentheses.

French sire	ALP F (%)	ALP M (%)	SAA F (%)	SAA M (%)	Total (%)
ITA	5,396 (5.01)	1,821 (1.69)	8,216 (6.25)	1,704 (1.29)	17,137 (7.2)
CHE	374 (1.33)	107 (0.38)	305 (1.05)	93 (0.32)	879 (1.54)
CAN	276 (0.48)	167 (0.29)	69 (0.18)	30 (0.08)	542 (0.58)
Total	6,046 (0.16)	2,095 (0.05)	8,590 (0.31)	1,827 (0.07)	18,558 (0.29)

Table 4 describes the number of animals that are both genotyped and present in at least two countries. There is some overlapping when counting animals across countries because French animals, for example, are present in more than two countries. The animal count ranged between 53 and 449 for Alpine breed and between 15 and 258 for Saanen breed. The number of genotyped animals used in several countries remains limited (less than 1% whatever the country) when compared to the native pedigree.

**TABLE 4:** Count of genotyped animals recorded in several countries for each national pedigree independently of the origin of the animals. The proportion of these animals compared to their native pedigree is represented in the columns %_native_pedigree_.

Native pedigree	ALP		SAA	
	# Animal	%_native_pedigree_	# Animal	%_native_pedigree_
ITA	449	0.66	248	0.3
CHE	97	0.38	15	0.06
CAN	53	0.67	35	0.83
FRA	388	0.02	258	0.02

Table 5 describes the average pedigree-based and genomic inbreeding observed for genotyped animals. For Alpine breed, the averaged pedigree inbreeding is close for Switzerland (0.019), France (0.022), and Italy (0.021) and lower in Canada (0.015). We observed different trends in Saanen with high inbreeding in Canada (0.048) and then in Italy (0.036), France (0.025), and Switzerland (0.015). The averaged genomic inbreeding is higher than pedigree inbreeding whatever the country for both breeds with differences (genomic – pedigree) from 0.023 (Alpine in Switzerland) to 0.101 (Alpine in Canada).

**TABLE 5:** Average and standard error of pedigree-based and genomic inbreeding within each country and breed for genotyped animals.

	ALP		SAA	
	Pedigree	Genomic	Pedigree	Genomic
ITA	0.021 (0.045)	0.073 (0.049)	0.036 (0.048)	0.127 (0.044)
CAN	0.015 (0.032)	0.116 (0.058)	0.048 (0.042)	0.095 (0.078)
CHE	0.019 (0.036)	0.042 (0.039)	0.015 (0.027)	0.078 (0.034)
FRA	0.022 (0.012)	0.084 (0.034)	0.025 (0.013)	0.121 (0.039)

### 3.2 Linkage Disequilibrium

**FIGURE 2:**
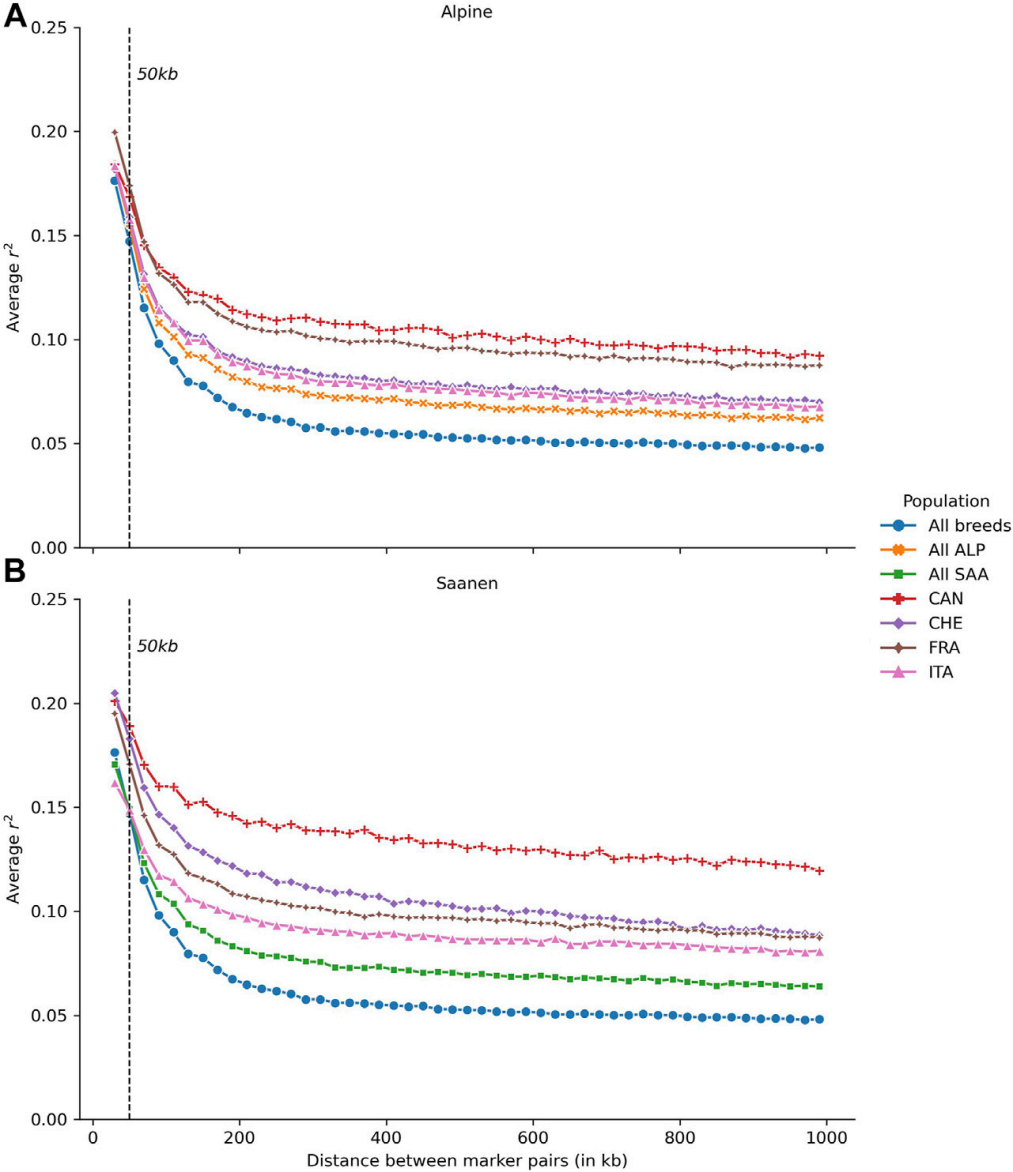
Average linkage disequilibrium (LD) in **(A)** Alpine (ALP) and **(B)** Saanen (SAA) breeds, according to the distance between SNPs for each country evaluated: Canada (CAN), Switzerland (CHE), France (FRA), and Italy (ITA) and Saanen from the four countries together (All SAA), Alpine from the four countries together (All ALP), and both Saanen and Alpine goats from the four countries (All animals).

The average LD calculated in Alpine (A) and Saanen (B), for each country separately and for multiple countries (ALP or SAA) or multiple breeds (All breeds) as a function of the SNP distance, is presented in Figure 2. For both Alpine and Saanen, the average LD was higher in Canadian than in the other goat populations. The average LD at 50 kb was 0.17 for Alpine and 0.19 for Saanen. The differences of LD values between Canada and the other countries were higher for the Saanen breed. For the Alpine breed, the average LD at 50 kb ranged between 0.16 (Italy) and 0.17 (France and Canada). The average LD was quite close between Canada and France, regardless of the distance between SNPs and the r^2^ values stabilized around 0.10–1 Mb. The average LD for the Swiss and Italian populations was also very similar and stabilized around 0.07–1 Mb.

For the Saanen breed, the range of LD values at 50 kb was wider than in the Alpine breed, with an average LD at 50 kb between 0.15 (Italy) and 0.19 (Canada). Canadian populations had a higher LD than in the other countries, regardless of the distance between SNPs. For short distances, LD values for Canadian and Swiss populations were close (0.18 and 0.19 at 50 kb, respectively) before differentiating for distances greater than 90 kb. The maximum difference was observed at 810 kb with an average LD of 0.09 in Swiss and 0.12 in Canadian goats.

### 3.3 Consistency of the Gametic Phase

**FIGURE 3:**
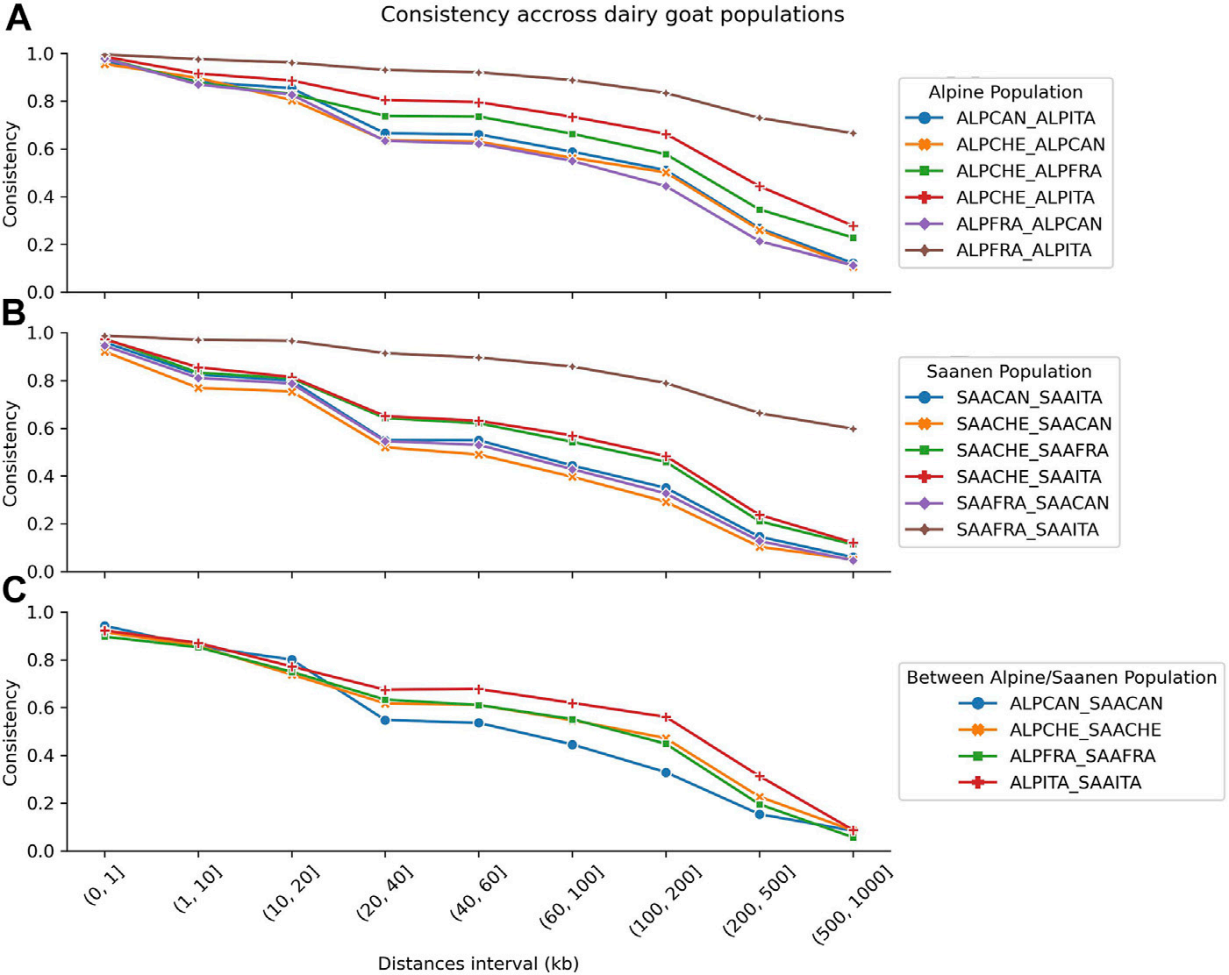
Comparison of the consistency of the gametic phase for nine classes of distances between SNPs with comparison between Alpine populations **(A)**, Saanen populations **(B)**, and Alpine and Saanen from the same country **(C)**. Breeds are represented by Alpine (ALP) and Saanen (SAA), while countries are represented by Canada (CAN), Switzerland (CHE), France (FRA), and Italy (ITA).

The consistency of the gametic phase according to nine classes of distances between SNPs is shown in Figure 3. Figures 3A,B present the consistency of the gametic phase between pairs of countries within the Alpine (A) and Saanen (B) breeds. Figure 3C presents the consistency of the gametic phase between the Alpine and Saanen breeds within each country. Within the Alpine breed (Figure 3A), the consistency of the gametic phase values was the highest between France and Italy and ranged from 1 (distance of (0, 1 kb]) to 0.67 (distance (500, 1,000 kb]). The lowest values were obtained when comparing Canadian and European populations (ALPCAN_ALPITA, ALPCHE_ALPCAN, and ALPFRA_ALPCAN). In this case, the consistency was on average 0.97 for a distance of [0, 1 kb] and dropped to 0.11 for a distance of [500, 1,000 kb]. The intermediate consistency of the gametic phase was obtained when comparing Switzerland to France or Italy (ALPCHE_ALPFRA and ALPCHE_ALPITA) with an average consistency of 0.97 for a distance of (0, 1 kb] and a drop to 0.17 for a distance of (500, 1,000 kb].

The trends observed in Alpine were also found in the Saanen breed (Figure 3B) but with slightly lower values than in the Alpine breed. Between France and Italy, the consistency of gametic phases varied between 0.99 for a distance of (0, 1 kb] and 0.60 for a distance of (500, 1,000 kb]. For a distance of (500, 1,000 kb], the consistency of the gametic phase values for all pairs of countries ranged between 0.06 and 0.60, while in Alpine, these values ranged from 0.11 to 0.67.

Figure 3C shows the consistency of the gametic phase within country when comparing Alpine and Saanen populations. The consistency of the gametic phase is similar for all countries for short distances with an average consistency of 0.92 for (0 kb, 1 kb], 0.86 for (1 kb, 10 kb], and 0.77 for (10 kb, 20 kb]. Then, the consistency between French and Swiss goat populations is similar across all distance intervals with an average difference of 0.01. The highest differences were observed between Canadian and Italian populations with an average difference of 0.10 across all distance intervals.

### 3.4 Principal Component Analysis

**FIGURE 4:**
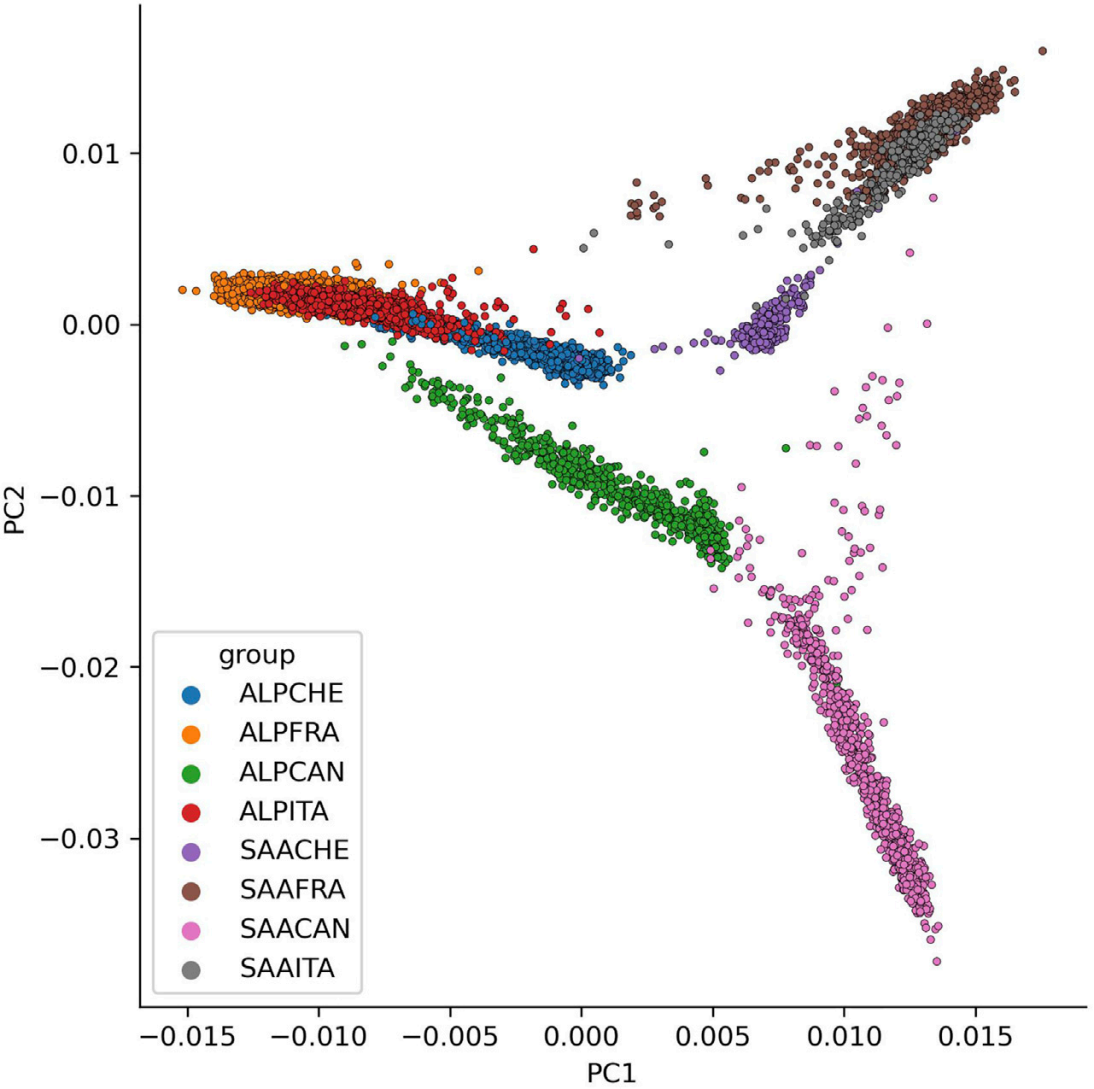
Principal component analysis (PCA) with all genotypes for each breed (ALP, Alpine; SAA, Saanen) and country (CAN, Canada; FRA, France; ITA, Italy; CHE, Switzerland) on the two first PCA components (PC1 to PC2).

Figure 4 presents the projection of each individual on the first two principal components of the PCA (PC1 and PC2). The first two components allow separate individuals according to their breed (PC1 3.26%), with the Alpine animals on the left and the Saanen on the right, and according to their country (PC2 2.32%), with the Canadian populations at the bottom and the European populations at the top. The French and Italian populations largely overlap and are indistinguishable for both breeds. The Canadian Saanen population is the most differentiated and does not group with the other Saanen populations. The few individuals present between the Canadian Saanen and the European populations are in fact animals with at least one French parent. The Swiss Saanen population is also more differentiated from the other European Saanen populations than the Alpine. Indeed, for the Alpine, there is an overlap of individuals for France, Italy, and Switzerland, which is not the case in Saanen with a more homogeneous cluster.

### 3.5 Admixture

**FIGURE 5:**
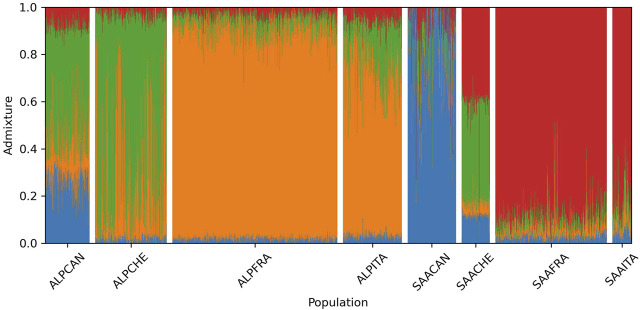
Breed composition per animal for each breed-country population estimated by the Admixture software when considering k = 4 (ALP, Alpine; SAA, Saanen; CAN, Canada; FRA, France; ITA, Italy; CHE, Switzerland).

The breed composition for each animal calculated with the Admixture software is shown in Figure 5. This analysis determines, for a given genotype, the proportion originating from each k ancestral cluster. The lowest cross-validation errors were observed when k was equal to 4. It was observed that the French and Italian populations have close and similar genetic background. For Alpine, on average, 0.89 of the genome of French goats and 0.72 of the genome of Italian goats come from the same ancestral cluster (orange color in Figure 5). This cluster is present to a lower extent in the Canadian (0.19) and Swiss (0.26) populations. On the other hand, there is very little present in Saanen (less than 0.10 for all populations). A second ancestral cluster (red color in Figure 5) is predominant in Saanen for French (0.88) and Italian (0.79) goats. This cluster is present at 0.41 in Switzerland for Saanen but is almost absent in Canadian Saanen (0.06).

The Canadian Saanen population seems to be largely different from the other Saanen groups. Indeed, the main ancestral cluster in Canadian Saanen (blue color in Figure 5) covers 0.82 of the genome, while it represents, on average, 0.11 for Swiss animals, 0.02 for French, and 0.04 for Italian animals. This blue cluster is also strongly represented (0.30) in the Canadian Alpine population. Another ancestral cluster (green color in Figure 5) also seems to be widely shared between Swiss Alpine (0.69), Canadian Alpine (0.46), and Swiss Saanen (0.41).

## 4 Discussion

### 4.1 Pedigree Connectedness

The connections between populations coming from the four different countries based on their pedigree information are an essential parameter for a successful international genetic evaluation, especially when using the single-step GBLUP method. On the other hand, to simplify the creation of a unified pedigree, it is important to have a unique identifier for each animal, which did not exist in goat populations in this study (and which is also rarely the case for cattle and sheep breeds). Here, some of the pedigree connections have been found, but there is still work to be carried out because some original pedigree of foreign animals is still untraceable. The importance and difficulty of exhaustive research of pedigrees have been discussed in previous studies, such as in beef cattle for Interbeef (Venot et al., 2007), dogs (Wang, 2018), and race horses (Viklund et al., 2015).

We also have disproportional datasets with larger amounts of data in France in comparison to the other countries. This situation has also been reported in the framework of Interbeef for the Limousin cattle breed (Bonifazi et al., 2020), in which the numbers of French animals (2,942,297 animals) were higher than in the other countries (between 30,843 and 172,229 animals). The authors evaluated the within-country rankings of the top 100 animals for age-adjusted weaning weight (AWW) for both international and national evaluations. They observed that the majority of the animals in the top 100 were French (between 84% and 100%) for the international evaluations, while they vary between 19% and 77% (100% being obtained in France) for the national evaluations. This is a situation that can potentially be reproduced in the international for dairy goat evaluations and could encourage the disproportional use of French breeding stock. Moreover, trade between countries has been mostly one-sided, with France exporting to all partner countries. Therefore, more research needs to be conducted to elucidate the best options for short- and long-term international and national genomic evaluations for the partner countries to maximize the benefits of the collaboration. In addition, genotyped animals represent only a small portion of shared animals between countries. Strategies for improving connections between countries need to be considered before implementing a multi-country genetic evaluation.

Inbreeding estimated in our population was also consistent with previous estimation found in the literature. For example, in Canada, Brito et al. (2017) reported an average pedigree inbreeding equal to 0.021 in Alpine (against 0.015 in our study) and 0.040 in Saanen (against 0.048 in our study). In France, Carillier et al. (2013) reported a pedigree inbreeding of around 0.02 for both Alpine and Saanen breeds which is consistent with our estimation. For Italy and Switzerland, it seems that no report was available that estimates inbreeding in these populations.

### 4.2 Linkage Disequilibrium and Consistency of the Gametic Phase

Population parameters such as LD and consistency of the gametic phase have implications for the design of across-population genomic evaluations. For a multi-population (here multi-country) genetic evaluation to be effective, there should be equivalent LD between SNPs and QTLs in each country and a relatively high consistency of gametic phases between populations from different countries (Mohammad Rahimi et al., 2020). For the French Saanen breed, the LD at 50 kb estimated in this study (0.19) is slightly higher than that observed in the study of Carillier et al. (2013) (0.17). In contrast, for Alpine, similar estimates were obtained (0.17 at 50 kb). The difference observed for Saanen can be explained by the difference in the numbers of animals used to calculate the LD values, which could impact the accuracy of the estimates. In the study of Carillier et al. (2013), the calculation of LD was determined for the Alpine breed on 470 Alpine genotypes compared to 2,968 in our study. For the Saanen breed, our study was based on 2,009 genotyped animals compared to 355 in Carillier et al. (2013). For Canada, the study by Brito et al. (2015) estimated the LD at 55 kb of around 0.14 for both breeds. We obtained higher values with 0.17 for Alpine and 0.19 for Saanen, which are identical to the estimates found for these breeds in France. Several factors can explain these differences in the estimates. The number of genotyped animals has increased substantially (403 vs. 793 Alpine and 318 vs. 903 Saanen), which contributes to obtaining more accurate estimates. On the other hand, although in both studies LD was estimated based on the r^2^ metric, the bins used to group the SNPs are different. Between 10 and 100 kb, Brito et al. (2015) created intervals of 10 kb, while we used wider intervals of 20 kb. To the best of our knowledge, no study has investigated the LD in Italy and Switzerland goat populations. Our study shows that Saanen populations from these countries have similar levels of LD in comparison to the French Saanen population. For the Alpine breed, the LD in Italian and Swiss populations is lower than in French Alpine. In any case, the level of LD is very close at 50 kb between populations and sufficient to consider genomic evaluation, as was demonstrated by Carillier et al. (2014). However, this level of LD will likely require larger training populations in comparison to less genetically diverse populations to obtain similar GEBV accuracies.

The consistency of the gametic phase is a key parameter for determining the effectiveness of a multi-population genetic evaluation (Biegelmeyer et al., 2016; Deng et al., 2019). This is the first time, to our knowledge, that the consistency of gametic phases is estimated between North American and European dairy goat populations. We observed that the French and Italian populations (Alpine and Saanen) have very high consistency of the gametic phase up to large distances between SNP pairs, indicating that a joint genomic evaluation might be feasible for these two countries. The consistency of gametic phase values is lower than the Canadian population with the European populations. This is also the case for Swiss when compared to French and Italian populations. This may make it more difficult to implement an international genetic evaluation across all the four countries. Deng et al. (2019) suggested that using a higher-density SNP chip panel could be an alternative for increasing the consistency of the gametic phase between SNP pairs (especially at shorter distances between SNPs). However, there are no high-density SNP chip panels available for goats. The availability of a second version of the Goat SNP50 BeadChip did not add enough SNP to get a significantly higher density of SNPs across all the goat genomes.

The consistency of the gametic phase in Alpine and Saanen breeds is similar within countries until the SNP distance of (10, 20 kb) with a decrease from about 0.92 to 0.62. After this distance, the decrease of the consistency of the gametic phase shows different trends with a higher level for Italian, a lower level for Canadian, and an intermediate level for Swiss and French populations. For French animals, these results are in accordance with those of Carillier et al. (2013), with a decrease from 0.88 to 0.56 for marker distance <50 kb vs. 0.89–0.63 in our study. For Canadian populations, Brito et al. (2015) reported a Pearson correlation of 0.69 at 20 kb between Alpine and Saanen breeds, which is also consistent with our study. Carillier et al. (2014) have shown that in the case of the French populations, multi-breed or single-breed genomic evaluations yielded similar GEBV accuracies. However, the number of genotyped animals was significantly smaller in their study. In the context of an international genomic evaluation, the interest of a multi-breed multi-country genomic evaluation will have to be evaluated in comparison to a single-breed multi-country evaluation, which could significantly increase the training population size per breed. However, the current genotypes provided by the partners are both unbalanced in number and in the years of birth of the animals. In particular, between Italy and Switzerland for both breeds, there is almost no overlap in the birth year of the genotyped animals. This study is, in fact, the first one that was carried out on such data for these two countries. Further analyses should be performed with larger genotyped populations to confirm our findings.

### 4.3 PCA and Admixture

The results of the PCA and admixture analyses contribute to determining the genetic relationship of the animals, including the breed and country of origin. The only populations with no clear distinction are French and Italian goat populations for both breeds. Italy is the country that imports most animals from France, which may explain the genetic proximity between these two populations. In contrast, the Canadian and European populations are more genetically distant. This might be explained by the little exchange of animals and the geographical distance that separates Canada and the European countries. Finally, these results are consistent with the results observed on the connections between countries based on pedigree information.

Several genetic diversity studies have been conducted in goats. In France, the study of Oget et al., 2019 was conducted on eight French goat populations, but it included a few genotypes of Alpine (45) and Saanen (38) animals. Our results, with more genotyped animals, confirmed what has been previously shown for these two breeds. The French Alpine and Saanen populations are genetically different. A second study performed by Brito et al. (2015) compared genotypes from Alpine (403 animals) and Saanen (318 animals) from Canada and found that these two populations are genetically different.

The comparison of Alpine and Saanen genotypes within one country is well-documented more than international comparisons of these breeds. Denoyelle et al. (2021) is one of the few examples of an international comparison, which was carried out as part of the VarGoats project (www.goatgenome.org/vargoats.html). This project sequenced goats of different breeds from all over the world including Alpine and Saanen from France, Italy, and Switzerland. They studied the phylogeny of these breeds using a neighbor-joining tree constructed with 100,000 SNPs. For the Italian, Swiss, and French populations, our results are in agreement with their study, where a close relationship between France and Italy (for both Alpine and Saanen) and a greater distance with the Swiss goat population were observed.

The first two components of the PCA represent less than 6% of the total variation, which is quite limited. Even if we observe two different clusters between European and Canadian populations, these populations seemed close enough in order to blend all genotypes and to analyze genotypes conjointly. Further investigations on differences along the genome between animals from different countries could be interesting to detect genomic regions specifically selected in each country.

### 4.4 Implications of the Results and Next Steps

This work aimed to combine and analyze pedigree information and genomic data from four countries. Our analyses showed that an international evaluation would be most beneficial to the European populations that are genetically closer. However, it is necessary to verify the impact of Canadian data into international genomic evaluations, especially if other European dairy goat populations are added such as Yorkshire dairy goats (Mucha et al., 2015). Yorkshire goats represent a composite population potentially more similar with Canadian dairy goats due to more similar crossbreeding events. Pedigree connectedness and genotype analysis remain the first step before implementing an international genomic evaluation. The following steps will be to combine and analyze the phenotypes commonly recorded among the different country partners of the project. The joint analysis of phenotypes, pedigree, and genotypes will enable the estimation of genetic and genomic parameters between breeds/countries that will be potentially used in future genomic evaluations.

## 5 Conclusion

The genetic diversity and pedigree analyses performed in this study showed that the French and Italian populations are both the most genetically connected and more genomically similar. On the other hand, for the Swiss and Canadian dairy goat populations, the genetic connections are limited to the importation of a few French animals. Besides, they are genomically more distant than the other populations. The genetic diversity and population connectedness between the studied populations indicate that an international genomic evaluation might be more feasible for French and Italian goats. Further studies will investigate the accuracy of genomic breeding values when combining the datasets from these four populations.

## Data Availability

The genotypes were produced by several companies in each of the partner countries. These companies share these genotypes for research collaboration. Within the framework of the SMARTER project, agreements have been signed to supervise the use and publication of these genotypes. These agreements specify that the genotype data must remain private. However, upon request and research collaboration, they can be shared for specific use in research projects. Requests to access these datasets should be directed to MT, marc.teissier@inrae.fr.
